# Circulating herpes simplex type 1 (HSV-1)-specific CD8^+ ^T cells do not access HSV-1 latently infected trigeminal ganglia

**DOI:** 10.1186/2042-4280-2-5

**Published:** 2011-03-15

**Authors:** Susanne Himmelein, Anthony J St Leger, Jared E Knickelbein, Alexander Rowe, Michael L Freeman, Robert L Hendricks

**Affiliations:** 1 Department of Neurology, Klinikum Grosshadern, Ludwig-Maximilians University, Munich, Germany; 2 Department of Ophthalmology, University of Pittsburgh School of Medicine, Pittsburgh, PA 15213 USA; 3 Department of Immunology, University of Pittsburgh School of Medicine, Pittsburgh, PA 15213 USA; 4 Trudeau Institute, Saranac Lake, NY 12983 USA; 5 Department of Molecular Genetics & Biochemistry, University of Pittsburgh, Pittsburgh, PA 15213 USA

## Abstract

**Background:**

Therapeutic vaccines can be designed to enhance existing T cell memory populations for increased protection against re-infection. In the case of herpes simplex virus type 1, recurrent disease results from reactivation of latent virus in sensory ganglia, which is controlled in part by a ganglia-resident HSV-specific memory CD8^+ ^T cell population. Thus, an important goal of a therapeutic HSV-1 vaccine would be to enhance this population.

**Methods:**

HSV-1-infected mice were treated with TAK-779 to block CCR5- and CXCR3-mediated CD8^+ ^T cell migration during both acute and latent infections. Additionally, HSV-1-specific CD8^+ ^T cells were transferred into HSV-1 latently infected mice to mimic the effect of a therapeutic vaccine, and their migration into trigeminal ganglia (TG) was traced during steady-state latency, or during recovery of the TG-resident memory CD8^+ ^T cell population following stress-, and corticosterone-induced depletion and HSV-1 reactivation from latency. Bromodeoxy uridine (BrdU) incorporation measured cell proliferation in vivo.

**Results:**

TAK-779 treatment during acute HSV-1 infection reduced the number of infiltrating CD8^+ ^T cells but did not alter the number of viral genome copies. TAK-779 treatment during HSV latency did not affect the size of the TG-resident memory CD8^+ ^T cell population. Transferred HSV-specific CD8^+ ^T cells failed to access latently infected TG during steady-state latency, or during recovery of the TG resident HSV-specific CD8^+ ^T cell population following exposure of latently infected mice to stress and corticosterone. Recovery of the HSV-specific CD8^+ ^T cell population after stress and corticosterone treatment occurred with homeostatic levels of cell division and did not require CD4^+ ^T cell help.

**Conclusions:**

Our findings are consistent with the notion that the CD8^+ ^T cells in latently infected TG are a tissue-resident memory (Trm) population that is maintained without replenishment from the periphery, and that when this population is disrupted, it recovers without proliferation or detectable recruitment of HSV-specific CD8^+ ^T cells from the blood. The compartmentalization of the HSV-specific CD8^+ ^memory T cell population in latently infected TG will complicate the design of therapeutic vaccines.

## Background

Primary herpes simplex virus type 1 (HSV-1) infections typically occur at mucosal surfaces, where the virus invades sensory neurons, is transported to neuronal nuclei in sensory ganglia and establishes a life-long latent infection. Latent infections are characterized by a lack of virion formation despite retention of viral DNA. However, reactivation from latency and anterograde axonal transport can result in pathologies ranging in severity from asymptomatic shedding to minor blisters and sores to blindness and potentially fatal encephalitis [1-6]. Recurrences of herpetic disease are associated with exposure to stressors known to compromise T cell function such as ultra violet irradiation, stress, cancer chemotherapeutic agents, and immunosuppressive drugs [1,7-9]. Exposure of HSV-1 latently infected mice to restraint stress increases serum corticosterone levels, diminishes the trigeminal ganglion (TG)-resident CD8^+ ^T cell population, compromises the function of the remaining CD8^+ ^T cells [9-11], and induces HSV-1 reactivation from latency [8].

Based on the apparent link between immunesuppression and HSV-1 recurrence, several groups have explored the role of T cells in controlling HSV-1 latency in mice, and they have determined that T-cells are necessary for maintaining viral latency [8,12]. CD8^+ ^T cells infiltrate the sensory ganglia during the primary infection of the cornea or skin and become closely associated with latently infected sensory neurons [13-15]. In the C57BL/6 mouse model, at least half of the CD8^+ ^T cells in HSV-1 acutely and latently infected trigeminal ganglia (TG) are HSV-specific as demonstrated by their specificity for a known immunodominant epitope on HSV-1 glycoprotein B (gB_498-505_) [15,16]. These gB_498-505_-specific CD8^+ ^T cells (herein referred to as gB-CD8) express an activated phenotype and form an immunological synapse with neurons; dispelling the notion that latent virus is ignored by the host immune system [14]. Moreover, the ganglion-resident memory gB-CD8 T cells can block HSV-1 reactivation from latency through release of interferon gamma (IFN-¿) or lytic granules [12,13,15,17]. Therefore, while incapable of clearing HSV-1 from the body, CD8^+ ^T cells can actively suppress viral reactivation.

Therefore sterilizing immunity to HSV-1 is not currently feasible, but therapeutic vaccines, which improve the size and quality of the TG resident CD8 population, could prevent HSV-1 reactivation from latency and rapidly control HSV-1 replication at peripheral sites when reactivation and shedding does occur. To this end immunization strategies designed to augment the existing immune response have been undertaken [18] and reviewed in [19,20]. Based on the work summarized above, the most effective therapeutic immunizations would increase memory CD8^+ ^T cell populations in the sensory ganglia, in peripheral lymphoid organs, and at sites of viral shedding.

The chemokine receptors CCR5 and CXCR3 have a prominent role in directing the migration of neutrophils and natural killer (NK) cells into sites of infection including HSV-1 infected corneas [21-23], but their role in directing CD8^+ ^T cells into the infected TG has not been clarified. Mice that are deficient in either of these receptors exhibit increased HSV-1 titers in their TG following corneal infection [21,22,24,25], but this likely reflects an effect on NK cell infiltration since HSV-1 replication in the TG is largely controlled by innate immunity prior to CD8^+ ^T cell infiltration [26]. Moreover, the increased viral load in the TG and the known functional abnormalities of CD8 T cells limits the utility of CXCR3^-/- ^and CCR5^-/- ^mice for assessing the role of these receptors and their ligands in regulating CD8^+ ^T cell infiltration into the TG [27-29]. An alternative approach is to treat mice with TAK-779 (a non-peptide inhibitor of both CCR5 and CXCR3) beginning after replicating virus is cleared from the TG and before CD8^+ ^T cell infiltration.

Recent data suggest that the CD8^+ ^T cell population in HSV-1 latently infected sensory ganglia may be compartmentalized and not replenished from the periphery, a feature ascribed to tissue resident memory (Trm) CD8^+ ^T cells [30,31]. Support for this concept comes from two types of studies. In one study the gB-CD8 memory population was maintained in TG, but lost from the lymphoid and other non-lymphoid tissues of IL-15^-/- ^mice, suggesting that the population can be maintained without replenishment from the periphery [32]. In a second study, HSV-1 latently infected dorsal root ganglia (DRG) from mice expressing different CD45 alleles were transplanted to different sites under the kidney capsule of non-infected mice [30,33]. Following transplant virtually all CD8^+ ^T cells in the DRG were lost and HSV-1 reactivated from latency. Interestingly, the CD8^+ ^T cell population in each DRG recovered with all CD8^+ ^T cells expressing the CD45 allele of the original DRG donor. Recovery of the CD8^+ ^T cell population in the transplanted DRG was antigen specific and required recipient dendritic cells and CD4^+ ^T cells, suggesting that the remaining donor CD8^+ ^T cells in the DRG were expanded through HSV-1 antigen presentation by infiltrating DC and with help from recipient CD4^+ ^T cells. There appeared to be little if any infiltration of CD8^+ ^T cells from the blood as this would have resulted in mixed CD45 allelic expression in the two DRG.

Here, we investigated the establishment and maintenance of the memory gB-CD8 population in infected TG at their true orthotopic site. Our data demonstrate a role for CCR5 and/or CXCR3 in the initial establishment of a CD8^+ ^T cell population in latently infected TG, but not in maintenance of the CD8^+ ^T cell population during viral latency. Our studies also establish a failure of HSV-specific CD8^+ ^T cells to infiltrate HSV-1 latently infected TG either during steady state viral latency or during recovery of the TG-resident CD8^+ ^T cell population following stress and corticosterone-induced diminution. These findings further establish the CD8^+ ^T cell population in latently infected sensory ganglia as representing compartmentalized Trm-like cells.

## Methods

### Mice and Virus Infection

Female C57Bl/6 (Thy1.1^-/-^) mice and C57Bl/6 expressing CD90.1 (Thy1.1^+/+^) were purchased from Jackson Labs (Bar Harbor, Maine). Mice transgenic for the HSV glycoprotein B specific T-cell receptor (gB-T) were a gift from Francis Carbone. The gB-T mice were maintained and used as homozygote for the gB-T gene and CD45.1 via sibling mating. The gB-T was also crossed onto Thy1.1^+ ^background by mating gB-T mice to Thy1.1^+ ^mice and back crossing them for four generations to yield a Thy1.1, gB-T homozygote.

The RE strain of HSV-1 used in these studies was generated purified and quantified as previously described [34]. Mice were anesthetized by intraperitoneal (i.p.) injections of 1.25 milligrams of ketamine hydrochloride and 0.125 mg of xylazine. Topical corneal infection was then performed on unconscious mice. Briefly, the eyes were scarred 16 times with 30-gauge needle in a crisscross pattern, and 3 ¿l of PBS containing 1 × 10^5 ^plaque forming units of HSV-1 RE was applied to the eye. All experimental procedures were reviewed and approved by the University of Pittsburgh Institutional Animal Care and Use Committee and conformed to the ARVO Statement for Use of Animals in Ophthalmic and Vision Research.

### TAK-779 Treatment

Where indicated, mice were treated subcutaneously with 150 ¿g TAK-779, a dose previously shown to effectively inhibit the function of CCR5 and CXCR3 in vivo [35]. Control mice received a subcutaneous injection of the vehicle (PBS).

### Isolation of Lymphocytes from Tissue

For the isolation of lymphocytes from the spleen, blood, lungs, TG and cornea mice were anesthetized with 2.5 mg of Ketamine and 0.25 mg xylazine. The anesthetized mice were then euthanized by exsanguination following intraperitoneal injection of heparin sulfate in PBS, and blood was collected for later analysis. The spleens were mechanically worked into single cell suspension. The cornea, TG, and lungs were dissected and digested with 42, 21, and 84 units, respectively of collagenase type I (Sigma-Aldrich, St. Louis, MO) in 100 ¿l of 5% FBS in RPMI. The digested tissue was then dispersed by pipette and passed through a 40 ¿m filter (BD Labware, Bedford Maine).

### Quantative Real Time PCR

Total DNA was isolated from tissue using Qiagen DNeasy spin columns (Qiagen, Valencia, CA) and quantified by spectophotometry. Viral DNA was detected using primers specific for the HSV-1 glycoprotein H (gH): forward primer (5'-CGACCACCAGAAAACCCTCTTT-3'), reverse primer (5'ACGCTCTCGTCTAGATCAAAGC-3') and probe [5'(FAM)TCCGGACCATTTTC(NFQ)-3'. The PCR reaction was carried out as previously described [36].

### Flow Cytometry

Staining of the lymphocytes with markers specific for T-cell associated markers was carried out using previously described methods and reagents obtained from BD Bioscience (BD Pharmingen, San Diego, CA) [34,36]. Briefly, VLA-4 (CD49b) and LFA-1 (CD11a) were stained with RI-2 and 2D7 clones respectively. Both antibodies were purchased from BD Pharmigen. In vitro cultured T-cell activation was quantified using the anti-CD44 clone IM7 and the anti-CD69 clone H1.2 F3, both purchased from BD Pharmigen. The CD4^+ ^and CD8^+ ^T cell populations were also identified as previously described by using the anti-CD8¿ antibody clone 53.67 and the anti-CD4 clone RM4-5 purchased from BD Pharmigen. Granzyme B staining was performed using the BD Bioscience intracellular staining kit and the GB II anti granzyme B antibody conjugated to APC (Invitrogen). The antibodies employed in these studies included: anti-Thy1.2 (CD90.2, clone 53.21), that was conjugated either to APC (BD Pharmingen) or PE-Cy7 (eBioscience); anti-Thy1.1 (CD90.1, clone OX-7) conjugated to either a FITC or a PE-Cy7 (BD Pharmingen); CD45.1 clone A20) conjugated to FITC; and CD45.2 (clone I04) conjugated to APC. The H2-Kb tetramers containing the immunodominant gB_498-505 _epitope were produced by the NIH Tetramer facility (Atlanta, GA) and used to quantify gB-specific CD8^+ ^T cells (gB-CD8). BrdU staining was carried out per manufacturer's instructions using the BD Pharmigen BrdU FITC Flow Kit (BD Pharmigen, San Diego, CA).

### In Vitro Activation of gB specific CD8^+ ^T-cells

Naive gBT-I transgenic spleen cells were coated for 1 h at 37°C with 1 ¿M gB peptide. Cells were then washed twice in HEPES-buffered Earles medium containing 2.5% (vol/vol) FCS before being cultured at a density of 1.7 × 10^5 ^cells per ml in complete medium (mouse tonicity RPMI 1640 medium: RPMI 1640 medium containing 10% (vol/vol) FCS, 2 U/ml IL-2 50 M -mercaptoethanol, 2 mM L-glutamine, 100 U/ml of penicillin and 100 g/ml of streptomycin). After 2 d, cells were washed and IL-2 was replaced with recombinant human IL-15 (20 ng/ml; R&D Systems). Complete medium containing human IL-15 was replaced every 3-4 d, and cells were used between 10 and 14 d after initiation of the culture [37].

### In Vivo CD4 Depletion, BrdU administration and Stress

Mice were depleted of CD4^+ ^T cells by i.p. injection of 0.15 mg of anti-CD4 antibody (clone GK1.5) at times described in the text. Stress was induced by restraining mice in aerated plastic tubes 11.5 cm long and 3 cm wide for 12 hrs, and was begun 2 hours after lights out to permit access to food and water prior to restraint. Mock stressed control mice were deprived of food and water for the same 12 hours, but were not restrained. After 12 hrs food and water was returned to all mice, but only stressed mice received corticosterone (0.4 mg/ml, Sigma-Alrdich, St. Louis, MO) in their water. After 4 consecutive stress treatments (referred to as T0) the mice were allowed to rest for the indicated amount of time without manipulation. When indicated mice received i.p. injections 1 mg/mouse of BrdU (Sigma-Alrdich, St. Louis, MO).

## Results

### Blocking the chemokine receptors CXCR3 and CCR5 reduces CD8^+ ^T cell infiltration into ganglia during acute HSV-1 infection

**Figure 1:**
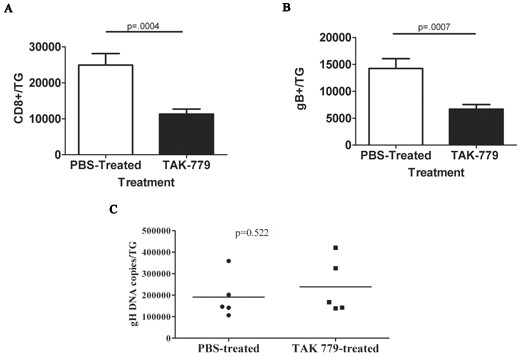
**Effect of systemic TAK-779 treatment on CD8^+ ^T cell infiltration into HSV-1-infected ganglia**. Six days following corneal HSV-1 infection, B6 mice were treated subcutaneously with 150 ¿g/mouse TAK-779 or with an equal volume of PBS. Two days following treatment, TG were excised and dispersed into single cells. The cells were stained for surface CD8¿, CD45, and gB-specific T cell receptor, and analyzed by flow cytometry. Data represent the mean ± standard error of cumulative data for total CD8^+ ^T cells **(A) **or gB-CD8 **(B) **per TG from three experiments involving a total of 6 PBS-treated and 11 TAK-779 treated mice. (**C) **DNA extracted from dispersed TG cells and analyzed by real-time PCR for the number of viral glycoprotein H (gH) gene copies revealed no significant group differences. A two-tailed Students t test was used for all statistical analyses.

Mice received a single subcutaneous injection of TAK-779 at 6 dpi to inhibit the activity of both CXCR3 and CCR5 [38] during the initial CD8^+ ^T cell infiltration of the TG that occurs 6-8 days after HSV-1 corneal infection. Total CD8^+ ^T cells and gB-CD8 were quantified by flow cytometry on dispersed cells from individual TG at 8 dpi. The TAK-779 treatment significantly reduced the CD8^+ ^T cell population in the TG compared to mock treated (PBS) controls, while not altering the frequency of gB-CD8 (Figure 1A&1B). The reduced CD8^+ ^T cell population in TAK-779 treated mice did not affect the HSV-1 viral burden in the TG (Figure 1C), which is largely controlled by the innate immune system prior to CD8^+ ^T cell infiltration [26].

### CXCR3 and CCR5 are not required to maintain the memory CD8^+ ^T cell population within latently infected TG

**Figure 2:**
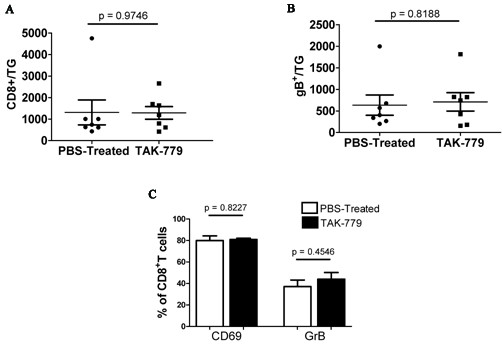
**Effect of TAK-779 treatment during HSV-1 latency on size and activation of memory CD8^+ ^T cell population**. HSV-1-infected mice were treated with TAK-779 or PBS at 30, 32 and 34 days post-infection (dpi). TG were excised 36 dpi, dispersed into single cells, stained for CD45, CD8¿, and gB-specific T cell receptor, total CD8^+ ^T cells **(A) **or gB-CD8 **(B) **per TG are presented. Data represent the means and standard errors for 9 PBS treated and 8 TAK-779 treated mice from 2 independent experiments. **(C) **Alternatively dispersed TG cells were stained for activation markers, CD69 and granzyme B (GrB). Data shown are the means and standard errors for 4 mice per group. Students t test revealed no statistical difference between the groups.

The CD8^+ ^effector T cells in the acutely infected TG undergo contraction and establish a stable memory population by 30 dpi [14]. To interrogate the role of CXCR3 and CCR5 in maintaining the memory CD8^+ ^T cell population in latently infected TG, mice were treated with TAK-779 or PBS as a control at 30, 32, and 34 dpi, and total CD8^+ ^T cells and gB-CD8 were quantified in the TG at 36 dpi. As illustrated in Figure 2, TAK-779 treatment did not influence the size of the total CD8^+ ^T cell population (Figure 2A) in the TG, or the frequency of gB-CD8 (Figure 2B). Thus, the chemokine receptors CXCR3 and CCR5 do not appear to be required to maintain the CD8^+ ^T cell population within HSV-1 latently infected TG, at least for the 6 day observation period employed.

CD8^+ ^T cells in latently infected TG of both mice and humans tend to cluster around latently infected neurons, some forming immunological synapses with the neurons, and most expressing an activated phenotype. Mechanisms governing the attraction of CD8^+ ^T cells to infected neurons and their activation status are unknown. Here we show that blocking CXCR3 and CCR5 with TAK-779 for 6 days did not alter the activation phenotype of TG-resident CD8^+ ^T cells (Figure 2C).

### HSV-specific CD8^+ ^T cells in the peripheral blood do not migrate into HSV-infected ganglia during viral latency

**Figure 3:**
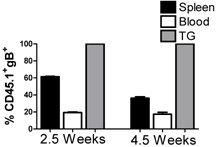
**Adoptively transferred gB-specific CD8^+ ^T cells are excluded from HSV-1-infected TG during stable latency**. CD8^+ ^T cells were isolated from 30 dpi gBT I.1 (CD45.2) spleens and 10^6 ^cells were intravenously (i.v.) injected into latently infected wild-type mice (CD45.1). At indicated times after transfer, spleens, blood, and TG were harvested and stained for CD8¿, CD45, CD45.1, CD45.2, and gB-specific T cell receptor. Data represent the percentage of gB-specific cells that are CD45.1+ (endogenous). Data shown are the means and standard errors for 3 mice at each time point.

It has recently been suggested that the CD8^+ ^T cell population within HSV-1-infected ganglia is compartmentalized, self-sustainable, and does not require replenishment from the circulating lymphocyte pool [39]. We employed adoptive transfer studies in which CD45.2^+ ^gB-CD8 memory or effector T cells were transferred to latently infected mice and their migration into the TG was monitored. In a first set of experiments gB-CD8 T cells were obtained from the spleens of CD45.2^+ ^gBTI1.1 mice 30 days after HSV-1 corneal infection, and transferred to latently infected CD45.1^- ^mice. The transferred and endogenous gB-CD8 T cells were quantified in the spleen, blood, and TG of the recipient mice 2.5 and 4.5 weeks after transfer. As illustrated in Figure 3, the gB-CD8 T cells in the spleen and blood of recipient mice contained an equal or greater frequency of donor cells at all times tested, but no donor cells were detected in the TG. Thus there is no detectable infiltration of HSV-1 specific CD8^+ ^T cells into the TG during stable latency.

### Repopulation of the TG following disruption via stress and corticosterone is not dependent on proliferation

**Figure 4:**
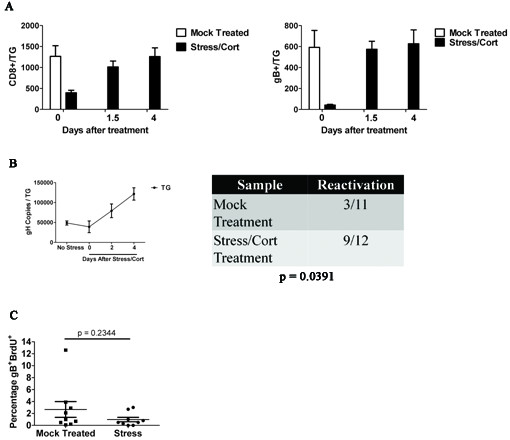
**Following disruption of the TG-resident CD8^+ ^T cell population via stress and corticosterone treatment, repopulation occurs independent of proliferation**. Latenly infected mice received 4 days of restraint stress and corticosterone-treated drinking water (400 ¿g/ml), followed by 1.5, 2, and 4 days of fresh water. TGs were harvested and stained with CD8¿, CD45, and gB-specific T cells receptor. **(A) **The numbers of CD8¿^+ ^cells and CD8¿^+^gB^+ ^were quantified via flow cytometry. Data shown are the means and standard errors for 7 (time point 0), 5 (time point 1.5), and 12 (time point 4) mice from two independent experiments. **(B) **RT-PCR of DNA from ganglia and corneas shows increased gH copies after stress and corticosterone treatment. gH copies peak in the TG at 4 days after stress and corticosterone while detectable viral burden in the cornea peaks at 2 days after fresh water (Fisher's exact test). RT-PCR of DNA from corneas shows increased detectable gH copies 2 days after stress and corticosterone treatment ended. TG data represents means and standard errors from 6 mice (No stress and day zero) or 2 mice (day two and four). **(C) **BrdU was given intraperitoneally (i.p.) 12 hours after fresh water. TG were then harvested 1.5 days after stress and stained for CD8¿, CD45, gB-specific T cell receptor, and BrdU incorporation. Data represent means and standard error of 9 mice from 2 experiments.

We previously established that exposure of latently infected mice to corticosterone or restraint stress reduces the CD8^+ ^T cell population in the TG, and exposure to restraint stress additionally induces HSV-1 reactivation from latency [8] (and Freeman and Hendricks, unpublished data). Here we show that simultaneous exposure of latently infected mice to stress and corticosterone on 4 consecutive days (30-34 dpi) reduced the CD8^+ ^T cell population in the TG by >80% (Figure 4A), but both the total CD8^+ ^T cell and the gB-CD8 T cell populations fully recovered within 4 days. It is interesting to note the original 1:1 ratio of gB-CD8 to gB-non-specific CD8^+ ^T cells was maintained following recovery of the population. Treatment also resulted in HSV-1 reactivation from latency as assessed by a significant increase in HSV-1 genome copy number in the TG and detection of HSV-1 genomes in the ipsilateral cornea (Figure 4B).

We hypothesized that recovery of the CD8^+ ^T cell population in the TG after stress and corticosterone treatment would involve proliferation of the remaining cells, infiltration of CD8^+ ^T cells from the blood, or a combination of both. We favored proliferation as re-establishing the original frequency of gB-specific CD8^+ ^T cells through infiltration from the blood would seem unlikely. However, the level of proliferation in the recovered population as assessed by BrdU incorporation was similar to that of the unperturbed population in mock treated mice that were not exposed to corticosterone and stress (Figure 4C), suggesting that recovery was not accomplished through proliferation of remaining cells.

### HSV-specific CD8^+ ^T cells in the peripheral blood do not migrate into HSV-infected ganglia following disruption of the TG-resident CD8^+ ^T cells

**Figure 5:**
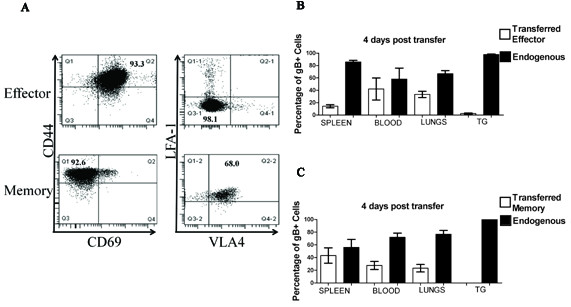
**Following disruption of the TG-resident CD8^+ ^T cell population via stress and corticosterone treatment, *in vitro *generated effector or memory cells do not enter the TG**. Spleens from naïve gBT I.1 Thy 1.1 mice were pulsed with SSIEFARL (gB_498-605_) and incubated in IL-2 for three days (effector cells) and some cells were further incubated for 10 days with IL-15 replacing IL-2 to generate memory cells. **(A) **Cells were stained for CD44, CD69, LFA-1, and VLA-4 and analysed by flow cytometry. Latently infected wild type mice (Thy1.2^+^) were subjected to 4 consecutive days of restraint stress and corticosterone. After the final treatment mice received an adoptive transfer of 10^6 ^effector **(B) **or memory **(C) **CD8^+ ^T cells (Thy1.1^+^). Four days after the adoptive transfer, spleens, blood, lungs, and TGs were harvested and stained for CD8¿, CD45, Thy1.1 (Donor), Thy1.2 (Recipient), and gB-specific T cell receptor. Data represent means and standard errors of the percentages of gB-CD8 that are of donor (Thy1.1) or recipient (Thy1.2) origin in the specified tissues (n = 4 mice per group). The experiments were repeated with similar results.

Therefore, we explored the alternative possibility that recovery of the CD8^+ ^T cell population in the TG following corticosterone and stress treatment was accomplished by infiltration of CD8^+ ^T cells from the blood. Memory and effector gB-CD8 T cells were generated *in vitro *by pulsing gBT-1 Thy 1.1^+ ^spleens with the gB peptide (SSIEFARL) in the presence of IL-2 for three days (effector cells), followed by culture with IL-15 for 10 days (memory cells) as previously described [37]. The effector cells were CD44^high ^CD69^+ ^LFA-1^low ^VLA-4^low ^whereas the memory cells were CD44 ^high ^CD69^- ^LFA-1^high ^VLA-4^high ^(Figure 5A). Latently infected Thy1.2 mice were exposed to stress and corticosterone for 4 days followed by adoptive transfer of effector or memory gB-CD8T cells. The TG, blood, spleens, and lungs were obtained 4 days after transfer, and the donor (Thy1.1) and recipient (Thy1.2) gB_498-505_-specific CD8^+ ^T cells were quantified. As illustrated in Figure 5B&5C the donor effector and memory cells comprised a significant portion of the gB-CD8 T cells in the spleen, blood, and lungs of recipient mice, but were undetectable in the TG. These findings do not support the concept that recovery of the CD8^+ ^T cell population in the TG is through infiltration of HSV-1 specific CD8^+ ^T cells from the blood.

### Repopulation of the TG following stress and corticosterone does not depend on CD4^+ ^T cells

**Figure 6:**
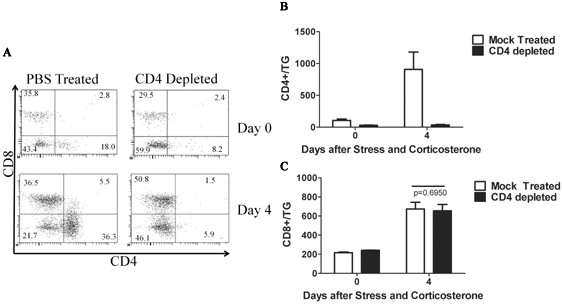
**CD4^+ ^T cells are not required for the recovery of CD8^+ ^T cells within corticosterone-treated ganglia**. Mice latently infected with HSV-1 received 4 days of restraint stress and corticosterone treatment (30-34 dpi). At 27 and 30 dpi mice received intraperitoneal injections of 0.75 mg/ml ¿-CD4 monoclonal antibody (clone GK1.5) or a control antibody. TG were excised at 38 dpi and stained for CD8¿, CD4, CD45, and gB-specific T cell receptor. **(A) **Represenative dot plots demonstrate effective depletion of CD4^+ ^T cells in the TG at 34 and 38 dpi. **(B) **Quantification of the CD4 depletion in mock depleted and CD4-depleted groups. **(C) **TG were excised at 38 dpi and analysed for recovery of the CD8^+ ^T cell population. Data are presented as means and standard errors of 15 (mock depleted) or 16 (CD4-depleted) mice from 3 independent experiments. Students t test revealed no significant differences in the number of CD8^+ ^T cells within TG from the mock depleted or CD4-depleted groups.

A previous study demonstrated that CD4^+ ^T cells are required for recovery of the CD8^+ ^T cell population in latently infected TG following transplantation under the kidney capsule of recipient mice [33]. To determine if this is also true for recovery of the CD8^+ ^T cell population in latently infected TG at the orthotopic site, latently infected mice were treated with stress and corticosterone on 4 consecutive days (30-34 dpi) and received anti-CD4 mAb on the first and last day of treatment. As shown in Figure 6A, the TG were completely depleted of CD4^+ ^T cells at the end of treatment and 4 days later (Figure 6B) when the CD8^+ ^T cell population had fully recovered. However, CD4^+ ^T cell depletion had no effect on recovery of the CD8^+ ^T cell population in the TG (Figure 6C).

## Discussion

The frequency of HSV-1 reactivation from latency in ex vivo TG cultures is inversely proportional to the size of the ganglionic CD8^+ ^T cell population [40]. Based on these findings some have advocated that all future HSV-1 vaccines be evaluated based on their capacity to enhance the HSV-specific CD8^+ ^T cell population in latently infected sensory ganglia. Such an approach would only be feasible if the HSV-specific CD8^+ ^T cells expanded in peripheral lymphoid organs through immunization have access to latently infected ganglia.

Evidence obtained in a model system in which latently infected DRG are transplanted under the kidney capsule of recipient mice suggest that HSV-specific CD8^+ ^T cells do not infiltrate latently infected DRG even when the resident CD8^+ ^T cell population is substantially disrupted [33]. However, a number of factors that may be unique to the transplanted tissue could have influenced CD8^+ ^T cell migration in that model. The authors suggested that the loss of CD8^+ ^T cells from the DRG following transplantation was likely due to death resulting from the trauma of transplantation. Such trauma could also induce other changes within the microenvironment of the latently infected ganglion such as alterations in the chemokine and cytokine milieu that could influence CD8^+ ^T cell infiltration. Moreover, revascularization of the transplanted tissue could influence T cell migration.

Based on these concerns we sought a more physiological model in which to examine the HSV-specific CD8^+ ^T cell infiltration into latently infected TG. We employed a model in which HSV-1 reactivation from latency is induced by exposure to psychological stress rather than trauma to the TG, depletion of the ganglion-resident CD8^+ ^T cell population is accomplished through exposure to corticosterone, and CD8^+ ^T cell migration into infected TG is examined at the orthotopic site.

Our findings strongly support the notion that the unperturbed CD8^+ ^T cell population in latently infected TG is maintained without detectable replenishment from the peripheral blood. We provide two types of evidence in support of this theory. We first identified chemokine receptors that are used to direct CD8^+ ^T cells into acutely infected TG and asked if blocking these receptors during latency would result in diminution of the CD8^+ ^T cell population in latently infected TG. The chemokine receptors CCR5 and CXCR3 are highly expressed on activated T cells and are expressed in HSV- acutely and latently infected mouse and human TG as are the corresponding chemokines CCL5 and CXCL10 [24,29,41-43]. However, a direct influence of CCR5 and CXCR3 on CD8^+ ^T cell infiltration into acutely or latently infected TG has not been established. Here we demonstrate that systemic treatment with TAK-779, a chemical non-peptide inhibitor of both CXCR3 and CCR5 [38] significantly reduced the infiltration of CD8^+ ^effector T cells into acutely infected TG, but did not influence the size of the CD8^+ ^memory T cell population within latently infected TG. These findings suggested that if the CD8^+ ^T cell population in HSV-1 latently infected TG is maintained through infiltration of CD8^+ ^T cells from the peripheral blood, the CCR5 and CXCR3 chemokine receptors do not appear to play an essential role in directing their migration despite the presence of their ligands in the TG.

More direct evidence that HSV-specific CD8 T cells do not migrate into latently infected TG came from the observation that adoptively transferred gB-CD8 that are retained in the blood, spleen, lymph nodes, and lungs of recipient mice over an extended period of at least 4.5 weeks are not detectable in the TG during the same period (Figure 3). The above findings strongly suggest that TG containing an established CD8^+ ^memory T cell population and latent HSV-1 are not permissive to CD8^+ ^T cell infiltration. We next asked if diminution of the resident CD8^+ ^T cell population through elevated serum corticosterone levels and reactivation of HSV-1 from latency through exposure of mice to restraint stress would change the microenvironment of the TG, rendering it permissive to CD8^+ ^T cell infiltration. However, adoptively transferred gB-CD8 T cells did not enter the TG from the blood even when reactivation and diminution of the TG-resident CD8^+ ^T cell population was induced by exposure of latently infected mice to stress and corticosterone (Figure 4).

A caveat to these findings is that the adoptively transferred CD8^+ ^T cells obtained from gBT1.1 mice may not accurately reflect the migratory capability of the endogenous gB-specific CD8^+ ^T cell memory population in the lymphoid organs and blood of the host. This seems unlikely given that neither effector cells generated through in vitro stimulation for 3 days with the gB_498-505 _peptide, nor memory cells obtained by incubating the effector cells for an additional 10 days in medium containing IL-15 were able to enter the TG. The transferred memory population expressed high levels of the LFA-1 and VLA-4 adhesion molecules typically used by CD8^+ ^T cells to enter infected tissue. Moreover, CD8^+ ^T cells that were isolated from spleens of HSV-1 latently infected mice (30 dpi) also failed to enter the TG during recovery of the CD8^+ ^T cell population following stress and corticosterone treatment (data not shown). These findings combined with observations made with transplanted HSV-1 latently infected DRG, and with vesicular stomatitis virus infected brains strongly suggest that once a Trm CD8^+ ^T cell population is established in nervous tissue, further infiltration of cells from the blood is effectively blocked [33,44].

Since infiltration of CD8^+ ^T cells from the blood did not seem to account for recovery of the CD8^+ ^T cell population in TG of stress and corticosterone treated mice, we concluded that recovery likely resulted from proliferation of the remaining cells. However, proliferation (based on BrdU incorporation into CD8^+ ^T cells) was the same or slightly lower in the recovered population when compared to the homeostatic proliferation of the CD8^+ ^T cell population in control mice that were not exposed to stress and corticosterone. While it is possible that a burst of proliferation was missed, the low level of gB-specific CD8^+ ^T cell proliferation measured from 24-36 hours after terminating stress and corticosterone treatment would not seem to account for the rapid recovery of the CD8^+ ^T cell population.

Interesting differences between our findings and those obtained with transplanted latently infected DRG deserve discussion. In our study, the kinetics of CD8^+ ^T cell recovery, the composition of CD8^+ ^T cell population (frequency of gB-CD8 T cells), and the size of the recovered population were all independent of CD4^+ ^T cell help (Figure 6). This is in contrast to the transplantation model where donor CD4^+ ^T cells were required for recovery of the donor CD8^+ ^T cell population in the DRG. Also differing in the two models is the kinetics of recovery of the CD8^+ ^T cell population. In our model recovery was complete 4 days after terminating stress and corticosterone treatment. In contrast, recovery of the CD8^+ ^T cell population in the transplanted DRG was negligible until 6 days after transplant, and peaked at 9 days after transplant. Finally the size of the recovered CD8^+ ^T cell population in our model closely approximated that in unperturbed control TG, while the recovered gB-specific CD8^+ ^T cell population in the transplanted DRG was approximately 17-fold higher than that observed prior to excision.

We believe these differences can be explained by differences in the two models and may be informative. The loss of CD8^+ ^T cells from the DRG following transplantation may reflect death of the CD8^+ ^T cells due to the trauma of DRG excision and transplantation as suggested by the authors of that report. Thus, recovery of the population would require proliferation of the surviving cells that were at very low levels. This could explain the delayed kinetics of recovery and the requirement for DC and CD4^+ ^T cell help. In contrast, corticosterone causes T cells to emigrate from tissues [45]. In our model, depletion of the CD8^+ ^T cell population from the latently infected TG may have resulted from migration out of the TG, followed by immigration back into the TG over the ensuing 4 days. This would explain the lack of proliferation of the recovered cells as well as the lack of requirement for CD4^+ ^T cell help. It would also explain the identical frequency of gB-specific CD8^+ ^T cells in the recovered and pre-treatment population. However, the failure of adoptively transferred cells to enter the TG during recovery would suggest selective re-entry of the original TG-resident CD8^+ ^T cells. This would suggest acquisition by the TG resident CD8^+ ^T cells of a homing receptor that permits selective re-entry into the latently infected TG. This possibility is under investigation.

Our findings suggest that at some point after establishment of an HSV-specific CD8^+ ^T cell population the infected TG becomes resistant to further T cell infiltration. We have shown that the gB-CD8 T cells exhibit a progressively higher functional avidity (ability to detect a low epitope density) over time in latently infected TG, whereas their counterparts in the spleen and lungs showed decreased functional avidity over the same period [46]. The ability to detect very low levels of MHC/peptide complexes on latently infected neurons would likely enhance the ability of CD8^+ ^T cells to provide immunesurveillance of latently infected ganglia, but be of lesser importance in the periphery. Therefore, there might be a selective advantage to the host to restricting infiltration of HSV-specific CD8^+ ^T cells into the TG. However, restricting entrance of CD8^+ ^T cells into the latently infected TG will complicate the development of therapeutic vaccines designed to bolster the TG-resident CD8^+ ^T cell population.

## Conclusions

Our findings suggest that augmenting the number of circulating HSV-specific CD8^+ ^T cells is not sufficient to bolster the HSV-specific memory T cell population in latently infected sensory ganglia. At some point after establishment of an HSV-specific CD8^+ ^T cell population the infected TG becomes resistant to further T cell infiltration. This might be an adaptation designed to prevent dilution of a highly functional CD8^+ ^T cell population that develops over time during latency with their less functional counterparts that are retained in lymphoid organs. However, compartmentalization of the memory population in latently infected ganglia will complicate the development of therapeutic vaccines designed to prevent HSV-1 reactivation from latency by increasing the size of the CD8^+ ^memory T cell pool.

## Competing interests

The authors declare that they have no competing interests.

## Authors' contributions

SH carried out TAK-779, adoptive transfer, and CD4 depletion experiments and aided in experimental design and analysis. AJS carried out adoptive transfer of effector and memory cells, BrdU, and CD4 depletion experiments and aided in experimental design, analysis, and drafting of the manuscript. AR performed BrdU experiments and helped draft the manuscript. JEK performed the PCR analyses and aided in experimental design, analysis, and drafting of the manuscript. MLF aided in psychological stress and TAK-779 experiments. RLH conceived the study, and participated in its design and coordination and helped draft the manuscript. All authors read and approved the final manuscript.
